# Risk Mitigation Measures Captured by a Tertiary Hospital's Disaster Simulation: An Observational Study

**DOI:** 10.1111/jep.70113

**Published:** 2025-05-05

**Authors:** Faran Shoaib Naru, Janet C. Long, Kate Churruca, Mitchell Sarkies, Jeffrey Braithwaite

**Affiliations:** ^1^ Centre for Healthcare Resilience and Implementation Science, Australian Institute of Health Innovation Macquarie University Sydney Australia; ^2^ School of Health Sciences University of Sydney Sydney Australia; ^3^ Implementation Science Academy University of Sydney Sydney Australia

**Keywords:** clinicians' disaster preparedness, clinicians' risk mitigation, hospital disaster simulation

## Abstract

**Aim:**

To document the challenges experienced and adaptations made during a simulated hospital disaster, and consider the implications of the observations for hospital disaster preparedness.

**Design:**

Nonparticipant observational assessment.

**Methods:**

Nonparticipant observations of an exercise simulating a disaster were undertaken by two researchers. The researchers shadowed triage team members, complementing this with observations of the Hospital‐Emergency‐Operations‐Centre, theaters, wards, and Emergency Department subsections such as Resuscitation, Acute, Minor‐Injuries‐Clinic, Children's emergency, and Mental health. Field notes were coded line‐by‐line through an inductive thematic analysis, which synthesized both challenges and observed adaptations to those challenges.

**Results:**

The major challenges observed were deaths due to lack of critical care equipment, management of high number of minor injuries, lack of situational awareness, shortage of orderlies, and difficulties in patient tracking and bed allocations. Observed adaptations included pediatricians' treatment of adult patients with minor injuries, fast‐tracking triage through ranking, manual ventilation during transfers, and batching of patients requiring imaging to utilize limited orderlies for transfers.

**Conclusion:**

This observational study distills both challenges that clinicians may face in real disasters, and the improvisations that they can make to manage mass casualties.

**Implications for Clinical Professions:**

Research findings hold promising potential in enhancing clinicians' disaster preparedness by articulating specific interventions on mass‐casualty management within limited resources.

**Impact:**

Unforeseen challenges arise when clinicians are confronted with disaster casualties. This study addresses that problem by not only preempting such challenges, but by also discussing practical solutions. The findings can enable a positive impact on clinicians' readiness for mass casualty influx.

**Reporting Method:**

The 21‐item checklist of the Standards for Reporting Qualitative Research (SRQR).

**Patient or Public Contribution:**

Although this study was not focused on a patient population, our research institute incorporates healthcare consumers' advice in all our work.

## 1 Introduction

### 1.1 Problem Formulation

Hospitals can enhance their preparedness by anticipating the challenges that clinicians may face in potential mass casualty events and by understanding how they will attempt to adapt. Unanticipated challenges can overwhelm responding clinicians, compromising their ability to deliver care to rapidly rising numbers of disaster patients, which may result in excess morbidity and mortality. This problem can be addressed by developing a deeper understanding of such challenges and how clinicians adapt their practices under pressure. Capturing prior disasters' lessons can overlook both the varied issues that arise as events unfold in a developing disaster and the improvisations that clinicians make in response to changing circumstances. Almost all studies on real disasters are retrospective analyses, which cannot always capture the fine details of different disruptions along with the adjustments, adaptations, and maneuvers that clinicians make to work around such disruptions. An exercise simulating a realistic disaster provides researchers a window into the real time dynamics that come into play when clinicians respond to a disaster scenario. A simulation can only approximate a disaster scenario, but it provides researchers with the best chance to estimate potential challenges of a real disaster. A simulation also provides an opportunity to prospectively understand the adaptations that can address such potential challenges, aiding exploration of additional resources that clinicians might need to respond well. Response related challenges overlooked by hospitals' disaster planning documents can potentially emerge in a real disaster or in an enacted response to an imagined one, and so proceedings of an exercise designed on a potentially real scenario can highlight important lessons for optimum disaster preparedness. Observation‐based research can capture the challenges faced by exercise participants along with their adaptations, and understanding such challenges and adaptations can enhance hospitals' preparations for real disasters.

Experiential learning or hands‐on learning in simulations has become an important part of hospitals' disaster readiness training. Various studies have provided evidence of the utility of simulations in improving participants' self‐assessed ability to respond to disasters [1, 2, 3, 4, 5, 6, 7]. Such disaster simulations are typically evaluated through surveys, conducted both pre‐post simulation exercise [1, 2, 3, 7], or exclusively post‐simulation [4, 5, 6], and look at participants' confidence in their ability to respond to a disaster, or perceptions of the utility of the exercise. A subset of research on simulation has evaluated participants' performance during the exercise against a predetermined criterion of avoiding preventable deaths and complications. This feature is built into Emergo Train System (ETS) exercise [8, 9]. ETS is a validated instructional tool that uses a bank of trauma patients to allow disaster responding organizations to simulate mass casualty events, and is used in Australia, United Kingdom, Netherlands, Italy, Japan, South Korea, Sweden, Finland, Greece, United Arab Emirates, and New Zealand [1, 3, 4, 5].

The simulation exercise observed by this study was designed by the hospital to assesses clinicians' ability to manage preventable deaths or complications [8]. Mortality and Morbidity Umpires were part of the exercise setup that researchers observed, and their role was to judge how clinicians managed critically injured patients or checked increases in disaster‐related morbidity under the pressure of mass casualty influx. This study did not duplicate the umpires' work by judging participants' performance. In a study on hospital disaster simulation with a research design of observational assessment [10], researchers scored participants' performance against a predetermined set of criteria. Our observational research did not assess the participants against any predetermined criterion. Rather, we adapted the methodology provided by Reeves and colleagues [11] which involved the researchers immersing themselves in the simulated setting to develop a rich understanding of clinicians' interactions, negotiations, and decisions. Observation of these dynamics can allow researchers the opportunity to collect critical data on the challenges that limited clinicians' ability to deliver care and how they adapted under those challenges. Such challenges can recur in real mass casualty events, so the focus of our study is on what exercise participants considered as important findings for future disasters. Instead of judging participants' performance or asking participants to judge changes in their own confidence levels, this study undertakes an arm's length assessment of issues and solutions that were observed in simulation and may recur in a real disaster. Such an observation‐based approach aimed at enhancing hospital disaster preparedness has not been adopted in prior research on hospital disaster simulations.

### 1.2 Purpose/Objectives

The objective of this study was to observe and synthesize challenges faced by participants during an exercise that simulated a realistic mass casualty disaster. An additional objective was to observe how clinicians adapted their practice under the pressure of mass casualty influx.

## 2 Materials and Methods

### 2.1 Qualitative Approach

Nonparticipant observations were conducted of a large‐scale disaster simulation exercise of a mass‐casualty event, undertaken in an Australian public tertiary hospital. The research output was reported according to the 21‐item checklist of the Standards for Reporting Qualitative Research (SRQR) [12].

### 2.2 Researcher Characteristics

Observations were made by two researchers, one from a clinical background and the other with disaster management expertise. Both health services researchers observed the entire proceedings of the whole hospital exercise, which simulated a grandstand collapse in a city on the east coast of Australia.

### 2.3 Context

The simulation exercise was conducted at one of Australia's largest tertiary hospitals. The 900‐bed tertiary hospital has over 6000 clinical staff, which includes approximately one and half thousand doctors and over 4000 nurses. According to the health services' website, this hospital had over 30,000 Emergency Department (ED) presentations and over 30,000 patient admissions in the first quarter of 2024. Over 200 hospital staff members participated in the 3‐h long simulation exercise on 29th November 2023. The scenario presented was a semi‐trailer accident resulting in a stadium grandstand collapse and explosion, which brought 120 patients to the hospital with crush, burn, smoke inhalation and other types of injuries. The exercise organizers used the ETS, which simulates over 1,000 patients using magnetic symbols that display patient data on the front and back (e.g., oxygen saturation level, respiratory rate, heart rate, blood pressure, Glasgow Coma Scale (GCS) reading, gender, age, and presenting conditions) [3, 13]. Exercise participants were able to attach stickers to the “patient” magnets representing clinical interventions such as intubation, intravenous fluids, oxygen supply, or blood transfusion. All interventions had associated times, which allowed exercise umpires to assess whether the cumulative mass casualty influx was managed under pressure. Participants in the triage subsection utilized the color‐coded triage system, which is prescribed in mass casualty management to quickly sort large number of patients into categories of walking wounded, injured but not critical, critical requiring lifesaving support, and unsalvageable or dead [14, 15]. These four categories are denoted by green, yellow, red and black stickers.

### 2.4 Sampling Strategy

The exercise was held in two locations of the hospital. Staff from the Intensive Care Unit, In‐patient wards, Children's Critical Care Unit, Neurology unit, Stroke unit, Medical day unit, Pediatric ward, Hospital Emergency Operations Centre, and all subsections of the ED including Triage, Resuscitation, Acute, Sub‐acute, Short Term Treatment Area, Minor Injuries Clinic, Children's emergency, and Mental health were observed in different areas of the hospital's Education and Pathology Building. A surgeon, anesthetist and their teams were observed in the hallway of their operating theaters.

### 2.5 Ethics and Governance Approvals

This study was developed and conducted in accordance with Australia's National Statement on Ethical Conduct in Human Research 2023 [16]. The tertiary hospital granted the governance approval, according to which, their Credentialing & Scope of Clinical Practice Committee granted both observers a limited Scope of Clinical Practice (SoCP) authorization for nonclinical observation.

### 2.6 Data Collection Methods

Before the start of the simulation exercise, the exercise organizers announced both researchers as nonparticipant observers. To develop an understanding of all stakeholders' interactions, negotiations and final decisions; both researchers observed all stakeholders, which included exercise participants, instructors, organizers, and the mortality and morbidity umpires. The umpires judged the participants against a predetermined criterion of addressing preventable issues in limited time, and so their judgments were also observed to capture any overlooked mortality and morbidity risks that disaster patients can face.

### 2.7 Data Collection Instrument

An observation tool, designed to capture information on clinicians' responses, adaptations and challenges as the simulation exercise unfolded, was developed by the first author (one of the observers), and reviewed and refined by the second observer. Besides allowing researchers to note clinicians' specific disaster response measures, the data collection tool also allowed observers to document broader sociocultural aspects of the simulation including the practice, social setting, culture, behavior, emotions, context, interactions, negotiations, and findings/conclusions of exercise participants [11, 17, 18]. The observers did not have any prior knowledge of the planned disaster setting or the challenges designed in the simulation exercise, therefore the observation tool was kept open‐ended and not tailored to a specific type of disaster.

### 2.8 Units of Study

Combinations of formal and informal observations were conducted by both researchers. Formal observations were conducted by shadowing the team of physicians and nurses that triaged almost all presentations, and informal observations were arbitrarily conducted by taking field notes on mass casualty management at all the different participant areas mentioned in the section on sampling strategy.

### 2.9 Data Processing

Both researchers separately made handwritten field notes aligned to the guidance in the data collection tool. They documented their observations throughout the disaster simulation and during the de‐briefing sessions; in which exercise participants provided their feedback and shared their own observations. As nonparticipant observers, both researchers did not talk to, or ask questions from, any of the observed participants and no electronic equipment was used to record or automatically transcribe any observations. Participant quotes were extracted from handwritten field notes and later tabulated and cross checked by both observers.

### 2.10 Data Analysis

The observers held a detailed preliminary discussion on emergent issues after the conclusion of the simulation in situ at the tertiary hospital. The observers debriefed each other to compare observations and resolve any discrepancies in the interpretation of events. Through these discussions, preliminary themes related to “lack of capacity to treat minor injuries” and “clinicians' frustrations at not being able to find beds for patient transfer” were identified and fed into the development of the coding framework. Both researchers later tabulated their handwritten field notes in Microsoft Word, leading to an expansion in the preliminary themes. The observers then triangulated their notes and through discussion agreed on additional overarching themes which included “orderlies' shortage” and “ineffective patient tracking”. The line‐by‐line coding process was then initiated by highlighting recurrent challenges and adaptations in the notes, and capturing them in overarching themes, either pre‐identified by both observers, or emerging from rigorous coding of the entire data set. To concentrate on unique themes emanating from the simulation, the thematic analysis did not code frequently implemented standardized disaster response measures, such as: coded announcement signaling a mass casualty event [19], elective surgery cancellations to manage patient loads [20], and setting up of incident command within hospital [21]. Researchers observed multiple instances of clinicians losing track of patients transferred by them or sent for imaging. All such coded observations were grouped together into the theme of ineffective patient tracking. Similar coding and thematic syntheses were undertaken for all themes. The inductive thematic analysis of both transcripts was conducted in NVivo 14 according to the analytical method of Kiger and Varpio [22] to synthesize the key risk reduction measures observed in the disaster simulation.

### 2.11 Techniques to Enhance Trustworthiness

Both observers triangulated their field notes according to the investigator/analyst triangulation method of Patton [23], to cross check the validity and reliability of collected data, and to limit any interpretive bias or selective perception bias in data collection. The triangulation generated deidentified observation notes for analysis. The field researchers refined the main themes through consensus.

## 3 Results

### 3.1 Synthesis and Interpretation

After the disaster was announced, the simulation exercise initiated multiple discharges of existing non‐disaster patients as participating clinicians anticipated mass influx of patients. A grandstand collapse was announced but no estimate of the patient load was provided, and neither were the injury types characterized. The patient load quickly built up, as exercise instructors kept inserting additional patients through the hospital's ambulance ramp. Patients with varying degrees of complexities were introduced and participants had to make quick collective decisions regarding their treatments or transfers based on their conditions and acuity. A challenging scenario was presented to participants to test what the exercise planners termed their ‘capability of interoperability’. During the exercise, the challenge was made more complex by introducing random disruptive events that could affect clinicians' ongoing responses. Two notable injects were the arrival of an unstable mental health patient requiring the support of security staff, and the town's mayor's phone calls to Hospital Emergency Operations Centre for updates.

**Figure 1:**
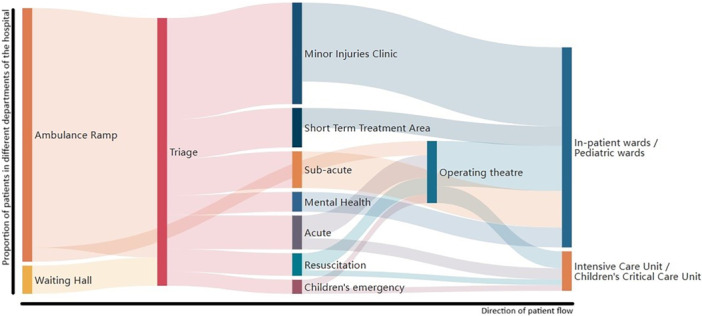
Approximation of patient flow during the disaster simulation.

Simulated ‘red blanket’ patients that might have severe trauma and blood loss were brought straight to the operating theater team, whereas all remaining disaster patients were routed through the triage subsection of the simulated ED. Figure 1 provides an approximation of the patient flow observed by researchers. The approximations in the figure are based on researcher estimations and not on specific patient numbers transferred from one space to another, as those numbers were unavailable. The researchers observed that after initial triage, most disaster patients presenting with minor injuries (some of whom made their own way to the hospital as ‘walk‐ins’) were allocated to the Minor Injuries Clinic, Short‐Term Treatment Area and the Subacute subsection of the ED, whereas relatively fewer numbers of critical patients were either taken directly to Operating Theaters as red‐blanket cases or were triaged to the Resuscitation, Acute, or Children's Emergency subsections of the ED. The In‐patient wards and Intensive or Critical Care Units received patients from the ED and Operating Theaters. The observers mapped the transfers in the following diagram which shows the approximated patient flow from left to right:

### 3.2 Links to Empirical Data

Through thematic analysis, the challenges of responding to a simulated disaster were identified. The identified challenges were ‘deaths due to lack of critical care equipment’, ‘management of minor injuries’, ‘lack of situational awareness’, ‘difficulties in bed allocations’, ‘ineffective patient tracking’, and ‘shortage of orderlies’. The thematic analysis also revealed how clinicians adapted to continue the delivery of care despite these challenges. The major adaptations were ‘effective management of limited resources’, ‘management of numerous presentations with minor injuries’, and ‘implementation of disaster triage’. The following sections elaborate major challenges and adaptations:

### 3.3 Challenges

#### 3.3.1 Deaths Due to Lack of Critical Care Equipment

Resource shortfalls characterized the simulated disaster as participants reported ‘blocked’ operating theaters, and high demands for imaging, ventilators and orderlies. The event generated a significant patient load requiring surgical interventions and imaging, to properly diagnose patients presenting with crush injuries. Although all 20 operating theaters of the tertiary hospital were functioning simultaneously, they proved insufficient for the number of critical patients. A participant in the acute subsection of the ED remarked “*operating theater is blocked and can take no more patients at present, so ED will need to hold onto surgical patients*” (Observer 2). Participants' diagnoses or risk stratifications were incomplete without imaging which had slowed due to high demand. At one point the pediatric or children's emergency subsection of the ED reported its patient statistics to Hospital Emergency Operations Centre as: “*1 red, 3 yellows, 3 walking greens, and 1 unknown because of delay in imaging*” (Observer 1).

The vulnerability of post‐disaster shortage of ventilators highlighted by the COVID‐19 pandemic [24, 25], was also observed in the simulated disaster. A participant from the Intensive Care Unit reported “*we are having to hand ventilate patients as there are no ventilators*” (Observer 1). Manual ventilation took clinicians away from attending other disaster affected patients as one participant from the Acute subsection of the ED noted: “*we ran out of ventilators, so our doctors and nurses were tied up for bagging*” (Observer 1). The participants from that subsection reported manual ventilation for extended periods. A participant from the pediatric or children's emergency subsection said: “*seven or eight children came that needed ventilating but there were no ventilators*” (Observer 2).

#### 3.3.2 Management of Minor Injuries

Several observed participants voiced the need for additional resources to treat minor injuries or conduct minor surgeries. These patients with minor issues formed the bulk of the total presentations. It was apparent that limited human resources and physical spaces were left for the majority presentations, those with minor injuries. The ED Resuscitation Bay was holding on to a lot of staff to have slack in the system so that it would be able to flex if more critical patients arrived. Such an approach seemed to minimize staff availability for other clinical tasks, including dealing with minor injuries. Patients needing major surgery were handled relatively efficiently at the Resuscitation and Acute subsections and the Operating Theaters, with multiple staff members allocated to their care. Meanwhile, those needing minor procedures were left till later.

In the Triage subsection, several subacute patients with nonlife‐threatening injuries denoted by green stickers started to accumulate. These patients with minor injuries used up resources in the Triage subsection and at one point staff members of the Triage subsection ran out of green stickers. A mortality and morbidity umpire, judging simulation participants against the predetermined criteria of addressing preventable deaths and complications, explained this in his debriefing:

“a lot of minor injuries could be attended, as minor casualties formed the bulk of the patient load, the largest number of interventions were needed in Minor Injuries Clinic, but there was no space in the hospital for patients that were not too serious to be sent to operating theaters. Spaces must be created for multiple day surgeries somewhere in the hospital so that the bulk of disaster patients could be treated without overwhelming the operating theaters.”

(Observer 1)

#### 3.3.3 Lack of Situational Awareness

We witnessed many clinicians who were unaware of the details of the simulated disaster and so could not adjust or change their practice according to the anticipated demands. The following observation illustrates clinicians' difficult juggling of limited beds, where they had inadequate situational awareness and lacked estimates of expected patients:

“There was uncertainty around how many disaster casualties were still to come and how many beds to save for them. In the ED, there was reluctance to fill up beds with less acute patients only to have to move them out if more serious ones arrived.”

(Observer 2)

The Hospital Emergency Operations Centre had the job of maintaining the big picture by collecting and collating the information, but top‐down communication was absent and coalface clinicians lacked the situational awareness needed to estimate the cumulative patient loads created by the disaster. An overview was needed for coalface clinicians to adapt their work according to anticipated bed demand. The Hospital Emergency Operations Centre acknowledged that they were not keeping up—or keeping the teams informed. The Hospital Emergency Operations Centre representative said: “*We didn't feel we had timely feeds but at the same time we were aware that we weren't telling staff what we did know*” (Observer 2).

There was also a lack of awareness across hospital departments as there was no dashboard presenting information such as bed availability in other hospital units or subsections. A participant from the Resuscitation subsection of the ED said: “*an oversight of resources in other subsections would have been really helpful for the responder needing or sharing resources*” (Observer 1). The Nursing Commander emphasized the need to know what was happening in the departments and all the subsections. Other participants also raised concerns about the lack of overview or updates. The clinicians strove to enhance their situational awareness as it was observed that the only way the team leaders or nursing commanders got an overview of the whole ED, and its bed situation, was by physically going and talking to the team leader of every other subsection.

#### 3.3.4 Difficulties in Bed Allocations

The exercise's participants were mandated to organize their patient transfers through a bed manager/incident controller. An exercise instructor explained to the participants: “*please book beds by calling the bed manager before sending any patients to a place, assuming that there would be beds there*” (Observer 1). Several observed participants expressed frustration at not being able to contact the bed manager or other departments for patients' transfers. Some participants complained that the bed manager's phone was always engaged. The team leader of the Subacute subsection of the ED said: “*can't get through to Patient Flow/Bed Managers as the line is constantly busy*” (Observer 2). The Nursing commander for ED remarked “*phoning for beds did not work*” (Observer 1). The team leader of the Acute subsection of the ED hung up the phone and said to his team: “*I've tried but they say they have no beds*” (Observer 2). Participants were unable to transfer their patients without reserving a bed with the bed manager, which consequently delayed care for other disaster patients queueing up at the hospital. Some participants discussed the need for an IT system as an alternative to difficult connections with the bed manager.

The participant executing the role of bed manager acknowledged the inability to respond to a large number of bed allocation requests from various departments. The bed manager said: “*the phone was constantly going, there were no dashboards available, so we had to ring up constantly to ask each area their bed status*” (Observer 2). Time and again our observation showed that the bed manager was inundated with phone calls and requests, which he was trying to catch up by calling back. The bed manager was overwhelmed with tasks which held up multiple patient transfers in other areas of the hospital.

#### 3.3.5 Ineffective Patient Tracking

Several clinicians complained about their inability to track the patients that they transferred or were supposed to receive. A participant from the in‐patient ward expressed frustration at their inability to track the patients that they were supposed to receive from the ED, if there were no beds left there. The participant indicated: “*are they in ED and about to come up or are there still beds there so there is no rush?*” (Observer 2).

While de‐briefing, participants explored solutions to the problem of ineffective patient tracking. A participant suggested electronic tracking: “*can we electronically track patients so we can keep track of where they go*” (Observer 2). A participant suggested a tracking admin officer if patient tracking dashboards would not be available. Another exercise participant remarked: “*do we need a map to track patients?*” (Observer 2). Some observed participants recommended solutions such as a wrist band with barcode to scan patients in and out of units.

The Hospital Emergency Operations Centre was tracking the progress of the entire simulation exercise, but they could not track the patients transferred from one place to another. A Hospital Emergency Operations Centre representative said: “*patient tracking would be a significant issue in a real disaster, so there should be a test of tracking system*” (Observer 1). In the simulation exercise, magnetic cutouts called ‘gubers’ symbolized patients and each guber had a unique patient number. A participant from the intensive care unit explained that tracking might be difficult in real disasters when multiple patients could have similar names. That participant said: “*patient tracking needed more thought … there would likely be a lot of John and Jane Does. It was relatively easy for us with numbered gubers*” (Observer 2).

#### 3.3.6 Shortage of Orderlies

Several participants expressed their displeasure at the shortage of orderlies, which held up their patient transfers, in turn delaying the care for other simulated patients. Participants could not transfer patients without orderlies, also known as ‘wardies’, and so had to wait for them to return from their last assignment. A participant said: “*bed cleaning and wardies coming back delayed things*” (Observer 1). A participant from the Acute subsection of the ED said “*we ran out of orderlies which held up transfers and taking patient to radiology, operating theaters etc*.” (Observer 2). The following observation illustrates how clinicians tried to adapt to the shortages of both the human resources such as staff needed to transfer patients, and the physical resources such as beds yet still maintaining safety and order:

“A patient with sore arm presented in ED. The patient was assigned a bed, then the bed was taken away as the patient could walk. The patient … (was) then planned to walk to the x‐ray facility themself. That led an exercise instructor to intervene who explained that patients must be accompanied by a staff member during transfer and cannot be allowed to walk around in the hospital by themselves because of the security risk that they can potentially pose.”

(Observer 1)

### 3.4 Adaptations

#### 3.4.1 Effective Management of Limited Resources

The participants found multiple solutions to work around resource shortages. The shortage of orderlies arose as a major challenge, as they were required to transfer patients or to take them to and from the imaging department. The simulated patient load of limb injuries required X‐rays to rule out orthopedic interventions. As there were not enough orderlies to take patients to the imaging department one by one, the participants started to group the patients that required X‐rays and then utilized one orderly to take a batch of patients for imaging.

Another shortage that carried significant mortality risks was that of ventilators. Critical patients were initially transferred from the ED to the wards with ventilators. The removal and delayed return of the ventilators to the ED denied that equipment to other critical ED patients. The Children's Emergency subsection of the ED at one stage had seven patients requiring ventilation without a single ventilator left in that subsection, as all had gone with patients to the Children's Critical Care unit. A representative from that subsection said: “*after a while (we) worked out it was better to send a doctor who could hand ventilate them and leave the ventilator in ED*” (Observer 2).

#### 3.4.2 Management of Numerous Presentations With Minor Injuries

As queues of patients requiring treatment of minor injuries kept building up, other subsections within the ED started delivering care to their immediate cohort. One observed example was the Children's Emergency subsection's acceptance of adult patients. The pediatricians assigned to the Children's Emergency subsection treated adult patients that could not be placed in the adult units due to a lack of staff and space. Two physicians participating in the exercise discussed: “*we need orthopedics and more doctors in the minor injuries clinic*” (Observer 1). An exercise participant from the ED asked:

“was it feasible to shift minor injuries normally handled by emergency department up to the wards to be managed there, for example small lacerations or un‐displaced fractures, but would need to work out who would take responsibility.”

(Observer 2)

A participant from the Pediatric ward said: “*we ended up using Room 5 to do minor procedures, which are not normally done there, but it worked out ok*” (Observer 2). Staff from Short‐Term Treatment Area, Subacute subsection, Pediatric subsection, and Minor Injuries Clinic tried to manage the bulk of patients with minor injuries. A team member from the Subacute subsection kept coming to Triage to ask for more patients and was observed to take five patients in one go. Team members from the Acute subsection also came up to the Triage team a few times to get more patients, and at one point only walked away with a green sticker minor injury patient requiring electrocardiography. A participant from the Short‐Term Treatment Area came up to the triage team and said that they had limited patients and indicated that wards, Intensive Care Unit and their Short‐Term Treatment Area were waiting for more patients.

#### 3.4.3 Implementation of Disaster Triage

The triage team adapted to the different disaster triage system by applying colored stickers to patient cutouts and ranking the patients. The triage team shared that they found themselves slipping back into standard triage but tried their best to adapt to the color‐coded disaster triage. The medical team leader of the Triage subsection said: “*the color‐coded triage is not very clear, as the nurses are too used to the normal triage and fall back to that every now and then because of familiarity*” (Observer 1). Another participant of the triage subsection remarked: “*triage worked well but it took time to get into the rhythm*” (Observer 2).

During the debriefing, participants discussed the switch to disaster triage. Many participants shared that the color coding was confusing, or patient severity was incorrectly interpreted or triaged. A participant remarked: “*there were patients that were triaged green that should have been red*” (Observer 1). Some participants voiced their preference for the usual or normal triage system (the Australasian Triage Scale) [26], while others noted that clinicians could triage the patients into a more urgent category after further assessment, in cases where they were incorrectly triaged. Participants highlighted the need to reevaluate the initial stratification made by the Triage subsection. After color coding their patients, the triage team delivered them to respective subsections, with reds going to Resuscitation or Acute, yellows going to Subacute, and greens going to Minor Injuries Clinic. Participants noticeably displayed initiative in addressing incorrect triage decisions. A participant from the subacute subsection of the ED said: “*we had the wrong person delivered to us, but we managed that*” (Observer 1).

The triage team also ranked their patients after color coding them, for quick transfers to other subsections, based on clinical needs. The medical team leader of Triage advised staff on this ranking of patients, following which a triage team member drew a column of the patient order. That team determined that if triage was overwhelmed, it would be best to use people for basic tasks.

## 4 Discussion

### 4.1 Integration With Prior Work, Implications, and Contributions to the Field

This study aimed to capture the challenges and adaptations observed in a tertiary hospital's disaster simulation. Multiple challenges were observed. Several exercise participants exhibited frustrations at not being able to make quick transfers because of bottlenecks in the system; for example, they could not find a bed in one of the wards, or they could not locate an orderly to go ahead with the transfer. Participants could not locate the patients that they transferred elsewhere, and they were not aware of the scale of the disaster to be able to effectively manage the discharges. Operating theaters were blocked, and so there was no space left for minor surgical procedures, which made up the bulk of the simulated presentations. Where feasible, clinicians adapted to address these challenges by engaging underutilized ED subsections for treatment of minor injuries and making judicious use of ventilators and orderlies.

Issues observed in simulated disasters can have significant implications for real disasters, and for that reason, besides giving an opportunity to play out the disaster plan, simulation exercises create opportunities for hospitals to update their plans [27]. In November 1993, a hospital in California, United States conducted a simulation exercise of hospital damage and a mass casualty influx due to an imagined earthquake, and 2 months later a 6.7 magnitude earthquake hit the hospital damaging glass windows, ceiling tiles, oxygen lines, power and telephone lines, and furniture, necessitating total evacuation [28]. Key learnings of the simulation informed the real evacuation, and 331 patients were safely moved out of the damaged hospital, with much of the success attributed to the simulation. Simulation exercises are expensive. All participants' roles need to be backfilled for the duration of their participation. Not all hospitals can afford such exercises, making it crucial to disseminate the learning of one hospital's simulation exercise to others. This study has endeavored to capture and disseminate some of those key lessons.

Existing research on simulation exercises is mostly limited to pre‐test post‐test surveys that report participants' self‐reported confidence in disaster response [1, 2, 3, 7]. Such studies fail to capture how clinicians adapt and negotiate under the stress of a simulated disaster. The observations undertaken in this study captured clinicians' actions and behaviors under pressure, their negotiations for patient transfers, and the way they handled minor injuries, treatment, and bed allocations. The high probability of similar negotiations being needed in real disasters requires prior understanding and identification of these key issues, delivered by this study. Observed adaptations can inform policy advice and suggest where specific strategies should be considered. This exercise showed, for example, that greater thought should be given to practical implications of manual ventilation during patient transfers, how to deal with the high number of walk‐ins with minor injuries, and how to increase undertaking of rapid triage of mass casualties.

An issue affecting the utility of the simulation exercise related to the low number of disaster patients with whom exercise participants were challenged. The hospital administration selected grandstand collapse over other disaster types. This selection created distinct challenges for a diverse cohort of participating clinicians. It generated a variety of minor and major injuries and issues requiring engagement of surgeons, anesthetists, pediatricians, ED consultants, nurses, ICU specialists, etc. Although simulated patients presented a range of complications justifying engagement of all types of clinicians, the number of patients was not enough to overwhelm the whole system, which is possible in other disaster types such as earthquakes, tsunamis, or cyclones. A total of 120 patients with varying needs were introduced to participants, whereas in some disasters, the number of injured can be in the thousands. Staff representatives from the Intensive Care Unit and the Children's inpatient unit immediately attended to ‘easy discharges’, freeing up beds for casualties. The Subacute subsection of the ED also repeatedly asked for more simulated patients. A limited patient influx meant that staff were not as challenged as they could have been from a scenario with very high numbers of casualties. The exercise seemed more about managing the existing resources at staff's disposal and less about managing insufficient resources in face of very high or even catastrophic need. Severe misalignment of needs and resources can be expected in a large‐scale disaster, but this was not a design feature of the exercise.

The exercise organizer explained at the outset that the scenario was designed to “test the plan and not the person.” It is assumed that this aspect of the simulation was emphasized to not let staff members be stretched to the limit, or to feel bad if any preventable mortalities were pronounced dead in the exercise due to a staff member's error under pressure, incorrect intervention or inability to attend to a case due to limited time. In real mass casualty incidents, clinicians would not necessarily have the comfort of feeling good about their actions or of being able to attend to all presentations, and neither would they be free of judgment. In the aftermath of Hurricane Katrina, the most researched of healthcare disasters [29], clinicians had to engage in litigation to absolve themselves of malpractice‐related charges following preventable deaths during evacuation [30].

This study highlights important implications of clinicians' extensive training to prioritize lifesaving work over treatment of minor injuries. Patients with minor injuries generally form the bulk of patient load, and their unchecked accumulation can create additional challenges for hospitals responding to disasters. Shortfalls of resources such as ventilators are now widely acknowledged as post‐disaster challenges [25]. Besides respiratory pandemics, disasters resulting in building collapse and crush injuries can also result in a shortage of ventilators, and this has been reported in some retrospective analyses of disasters. For example, the collapse of major infrastructure in 2010 Haiti earthquake resulted in the unavailability of ventilators for several children and neonates requiring critical respiratory support. The shortage was managed through the provision of a single ventilator in a field hospital [31].

This study has also underscored a generalizable experience, that coalface clinicians require data e.g., a thorough understanding of the disaster, estimated number of its casualties and types of injuries, and what is going on elsewhere in the hospital; to adjust their practice and make informed decisions, before and during the arrival of disaster patients. Although information technology‐based patient tracking systems can be vulnerable to disaster disruptions to power and connectivity, they can provide the most effective big picture or situational awareness that clinicians need to manage all disaster casualties. Our study reinforced the implications of ineffective patient tracking, also reported in a retrospective post‐disaster study of the 2011 earthquake and tsunami in Japan [32]. The challenges reported in this study are known to have arisen in prior disasters, so, inevitably, the reported adaptations here will be generalizable to clinicians' responses to future disasters. Research findings may also be beneficial for smaller or regional hospitals that always have limited supply of beds, critical care equipment such as ventilators, and essential personnel such as clinicians and orderlies. Smaller regional hospitals are also prone to post‐disaster communication disruptions which can preclude their clinicians' situational awareness. Tertiary hospital clinicians' adaptations captured by this study can therefore be seen as important lessons for all hospitals, big or small.

### 4.2 Limitation

A limitation of this kind of research is that the observers' presence can affect the actions being observed [33]. Researchers addressed this limitation through passive observation, i.e., not engaging in any conversations with participants and minimizing the impact of their physical presence.

## 5 Conclusion

The observational assessment of a disaster simulation exercise at one of Australia's largest tertiary hospitals facilitated the identification of important challenges that clinicians can expect to face in real disasters, if left unaddressed. The observations also identified clinicians' adaptations in disaster scenarios. Through the identification of these challenges and adaptations, we endeavored to provide empirically backed recommendations for improvements in hospitals' disaster preparedness plans.

## Author Contributions

Faran Shoaib Naru conceptualized the research, and with Janet C. Long, Kate Churruca, Mitchell Sarkies, and Jeffrey Braithwaite designed the research. Faran Shoaib Naru and Janet C. Long developed the data collection tool and refined the data collection methodology. Faran Shoaib Naru and Janet C. Long collected data through observations at the tertiary hospital disaster simulation. Data analysis and initial write‐up were done by Faran Shoaib Naru. Kate Churruca, Janet C. Long, Mitchell Sarkies, and Jeffrey Braithwaite refined the manuscript through multiple revisions. All authors developed and approved the final manuscript.

## Ethics Statement

This study was developed and conducted in accordance with Australia's National Statement on Ethical Conduct in Human Research 2023 (NHMRC 2023). The ethical approval for this study was granted by the Medicine and Health Sciences Subcommittee of Macquarie University's Human Research Ethics Committee: Reference No: 520221213642123, Project ID: 12136. The tertiary hospital granted the governance approval, according to which, their Credentialing & Scope of Clinical Practice Committee granted both observers a limited Scope of Clinical Practice (SoCP) authorization for nonclinical observation.

## Conflicts of Interest

The authors declare no conflicts of interest.

## Data Availability

The deidentified primary data collected for this study can be requested from the corresponding author.
